# Long-term Effects of the COVID-19 Pandemic on Public Sentiments in Mainland China: Sentiment Analysis of Social Media Posts

**DOI:** 10.2196/29150

**Published:** 2021-08-12

**Authors:** Hao Tan, Sheng-Lan Peng, Chun-Peng Zhu, Zuo You, Ming-Cheng Miao, Shu-Guang Kuai

**Affiliations:** 1 School of Design Hunan University Changsha China; 2 Emergency Science Joint Research Center Hunan University Changsha China; 3 Shanghai Key Laboratory of Mental Health and Psychological Crisis Intervention Key Laboratory of Brain Functional Genomics (Ministry of Education), The Institute of Brain and Education Innovation School of Psychology and Cognitive Science, East China Normal University Shanghai China; 4 NYU-ECNU Institute of Brain and Cognitive Science New York University Shanghai Shanghai China

**Keywords:** COVID-19, emotional trauma, public sentiment on social media, long-term effect

## Abstract

**Background:**

The COVID-19 outbreak has induced negative emotions among people. These emotions are expressed by the public on social media and are rapidly spread across the internet, which could cause high levels of panic among the public. Understanding the changes in public sentiment on social media during the pandemic can provide valuable information for developing appropriate policies to reduce the negative impact of the pandemic on the public. Previous studies have consistently shown that the COVID-19 outbreak has had a devastating negative impact on public sentiment. However, it remains unclear whether there has been a variation in the public sentiment during the recovery phase of the pandemic.

**Objective:**

In this study, we aim to determine the impact of the COVID-19 pandemic in mainland China by continuously tracking public sentiment on social media throughout 2020.

**Methods:**

We collected 64,723,242 posts from Sina Weibo, China’s largest social media platform, and conducted a sentiment analysis based on natural language processing to analyze the emotions reflected in these posts.

**Results:**

We found that the COVID-19 pandemic not only affected public sentiment on social media during the initial outbreak but also induced long-term negative effects even in the recovery period. These long-term negative effects were no longer correlated with the number of new confirmed COVID-19 cases both locally and nationwide during the recovery period, and they were not attributed to the postpandemic economic recession.

**Conclusions:**

The COVID-19 pandemic induced long-term negative effects on public sentiment in mainland China even as the country recovered from the pandemic. Our study findings remind public health and government administrators of the need to pay attention to public mental health even once the pandemic has concluded.

## Introduction

The COVID-19 outbreak has spread rapidly across the world, leading to a global pandemic. At the end of 2020, more than 82,662,478 confirmed cases of infection and at least 1,872,802 deaths were reported globally [1]. To prevent the spread of the pandemic, many countries imposed different levels of restrictions in their administrations, such as enforcing statewide lockdowns, home isolation, and travel bans. COVID-19, similar to previous infectious disease outbreaks such as SARS (severe acute respiratory syndrome) in 2003 and Ebola virus disease in 2014, has not only threatened the physical health of the public but also imposed a wide range of negative emotions, including fear, depression, and panic disorder [2-4]. Such negative emotions could be harmful to the public's mental health and even trigger social unrest [5]. Therefore, understanding how the pandemic affects public sentiment can provide valuable information for policymakers, government administrators, and mental health service providers.

Since the onset of the pandemic, researchers have conducted online and offline surveys to assess the public’s mental health. These surveys have consistently shown that the COVID-19 outbreak has had a devastating negative impact on the public’s mental health [6-9]. Meanwhile, with the growing popularity of the internet, people have extensively expressed their emotions through web-based platforms such as microblogs. The development of natural language processing allows us to identify and quantify people's emotional states by analyzing the content of their posts on the internet. The average emotional state of the entire community is defined as the *public sentiment*. Sentiment analysis of social media posts has been used in many previous studies and is believed to be a good indicator of public emotions in the internet era. Such sentiment analyses have indicated a significant increase in negative emotions and a decrease in positive emotions and life satisfaction [10,11]. Analyzing queries in search engines has also identified an increase in topics related to anxiety, negative thoughts, sleep disturbances, and even suicidal ideations [12,13]. Upon combining the evidence of studies by using different approaches, the negative impact of the outbreak on public mental health is unquestionable. However, it remains largely unknown how public sentiment has changed across various stages of the pandemic, such as the accelerating, decelerating, and “the new normal” stages.

**Figure 1:**
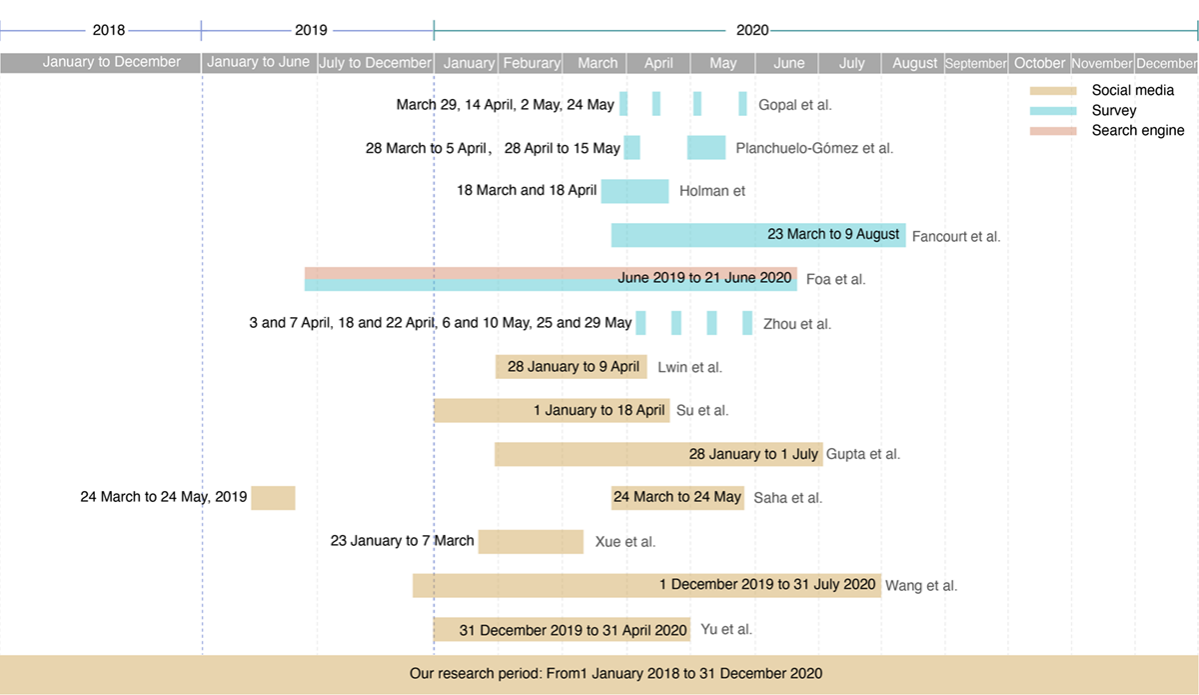
A literature map summarizing published studies on tracking public mental health during the COVID-19 pandemic. The horizontal axis labels the tracking time and different shading colors represent varying approaches to assess mental health.

Several tracking surveys have found an increase in negative emotional rating scores following the COVID-19 outbreak, as illustrated in Figure 1. These include Gopal et al in India [14], Planchuedlo-Gomez et al in Spain [15], Holman et al in the United States [7], among others. However, a literature search has yielded some contrasting findings. Fancourt et al [6] and Foa et al [12] found that negative emotional rating scores were the highest at the beginning of the outbreak and gradually decreased thereafter. Zhou et al [16] found a slight improvement in mood after the unlock phase of Wuhan, China, was initiated, by comparing residents’ mental health before and after the city’s reopening [16]. Moreover, studies using content analysis of social media and search engines showed the highest concerns and negative emotions to outbreak-related topics, and there was a gradual decrease in such negative emotions thereafter, as reported by Lwin et al [17], Su et al [11], Gupta et al [18], Saha et al [19], Xue et al [20], Wang et al [21], and Yu et al [22] (Figure 1).

Most studies thus far have focused on the psychological impact of the pandemic in the first half of the year, and neither surveys nor social media sentiment analyses have assessed the public sentiment after August 2020 to date. The long-term effects of the pandemic on public sentiment, therefore, remain largely unknown. As mainland China was the first region hit by the COVID-19 pandemic, and it has been the first major economy to successfully recover from the pandemic shock [23,24], monitoring public sentiment on social media in mainland China at different stages of the pandemic would provide a comprehensive understanding of the pandemic’s effect on public mental health. In this study, we tracked posts across Sina Weibo—the largest microblogging social media platform in China [25], which is analogous to Twitter worldwide. We analyzed the public sentiment reflected in Weibo posts across 2020 and identified interesting short- and long-term emotional trauma induced by the COVID-19 pandemic among Weibo users.

## Methods

### Collection of Weibo Posts

We used a web crawler technology to collect 64,723,242 Weibo posts from 26,895,593 accounts across 31 provinces, from January 1, 2018, to December 2020 (n=10,072,413 in 2018; n=21,652,707 in 2019; and n=32,998,122 in 2020). We tried to maintain uniform sampling across provinces to balance regional differences, with the number of posts published in each province ranging around 3000 posts per day in 2020, 2000 posts per day in 2019, and 1000 posts per day in 2018.

We calculated the number of Weibo posts per account. The data follow the Zipf distribution, which aligns with the results of Li et al [26]. A small number of users generated a very high number of posts; these are likely to be advertising accounts. We filtered out 0.01% of accounts with the highest number of posts and retained the accounts that were within 99.99% of the distribution of the Weibo post per capita. Moreover, accounts that were "verified" as those of stars, public figures, organizations, etc, were filtered out. Posts that appeared to be advertisements for marketing purposes were excluded as well. Additionally, we filtered out the posts that did not contain Chinese characters and those that exceeded 140 characters. The study was approved by the ethics committees of the East China Normal University and School of Design, Hunan University.

### Calculation of Sentiment Values

We applied the Tencent natural language processing product [27], a professional Chinese sentiment analysis application programming interface (API), to analyze public sentiment on the internet [28]. The algorithm excluded numbers, punctuations, English characters, URL, hashtags, mentions, and emojis and then extracted Chinese characters, numbers, and punctuation for sentiment analysis. The algorithm generated a sentiment value score ranging from 0 (extremely negative mood) to 1.0 (strongly positive mood). We averaged mean sentiment values for all 31 provinces in mainland China.

### Data of Reported COVID-19 Cases

We obtained the data on COVID-19 cases reported in mainland China from the National Health Commission of the People’s Republic of China and the health commissions of municipal provinces [29]. We collected data regarding global cases of COVID-19 from the World Health Organization and Johns Hopkins Coronavirus Resource Center [1,30].

### Data of Economic Indicators

We obtained data of economic indicators from the National Bureau of Statistics [31], including the consumer price index, unemployment rate in urban areas of mainland China, producer price index, and growth rate of gross domestic product (GDP). The first three indicators were calculated per month and the GDP growth rate was calculated per quarter.

## Results

### Social Media Public Sentiment Values Throughout 2020 and in the Previous Two Years

**Figure 2:**
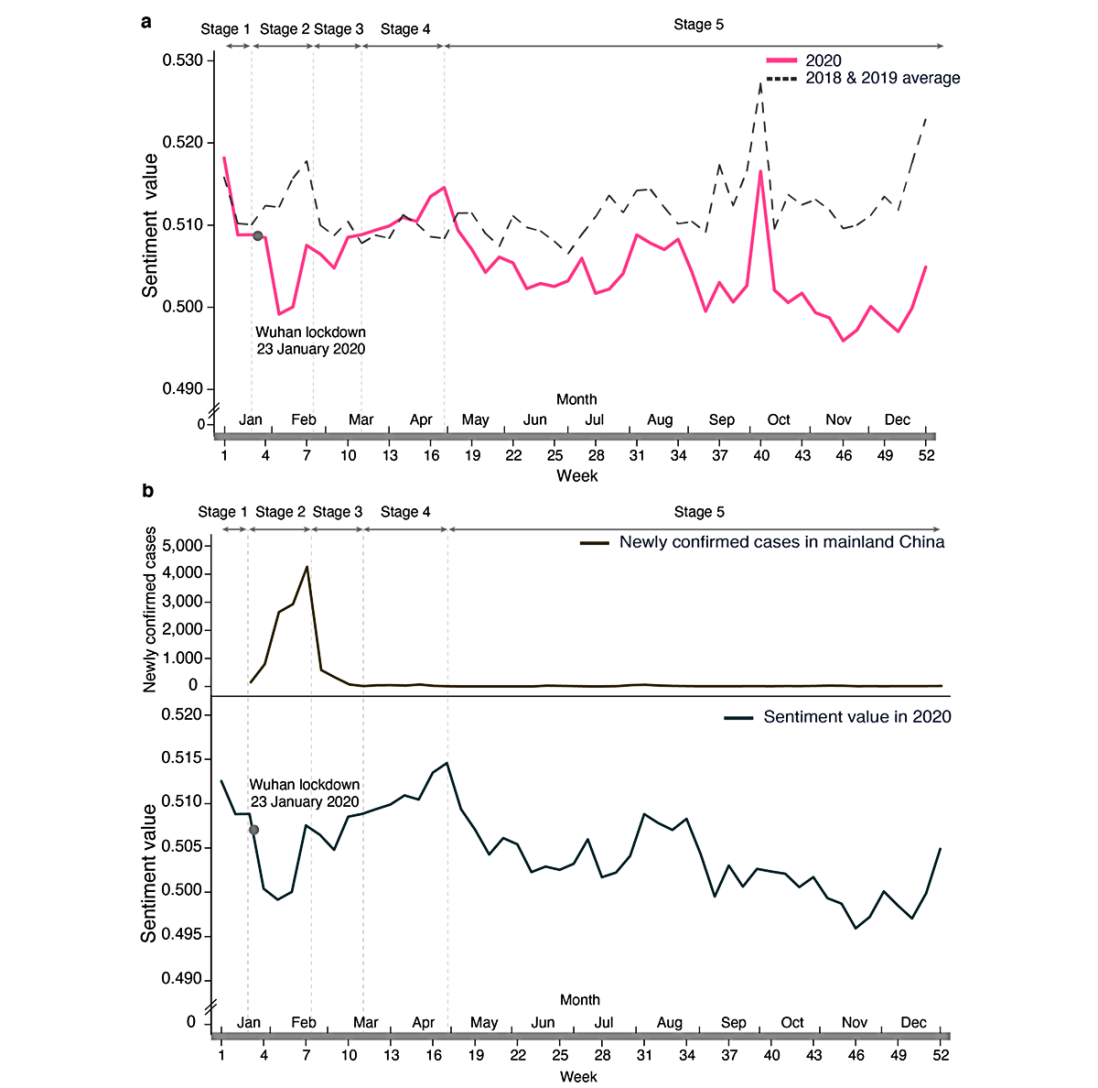
Public sentiment values on the Chinese social media platform Weibo over the last 3 years. (a) Public sentiment values were plotted as a function of the week across the whole year (2020) or averaged values of the previous 2 years. (b) The number of newly confirmed COVID-19 cases per week for 2020 (upper panel) and sentiment values in 2020 excluding holiday effects (lower panel).

In week 3 (January 20, 2020), human-to-human transmission of the virus was confirmed and announced to the public. Thereafter, the city of Wuhan entered a lockdown on January 23, 2020 [32], attracting considerable public attention. As a consequence, social media public sentiment values rapidly decreased, hitting the lowest in week 5 (Figure 2a). The lowest sentiment value was reduced by more than 3.7% compared to the beginning of the year (week 1). The sentiment values bottomed out from week 6 and reached the peak at week 17 (Figure 2a). Thereafter, sentiment values surprisingly entered a long-term downward spiral until the end of the year, although the pandemic was well under control in mainland China. The lowest sentiment value (mean 0.496, SE 0.002) in the second half of the year was even lesser than that in week 5 (mean 0.499, SE 0.003), marking the lowest point after the COVID-19 outbreak in stage 2 (Figure 2a).

It is unclear whether the decline in sentiment values in the second half of 2020 presents a long-term effect of the pandemic or whether they are merely seasonal fluctuations. To answer this question, we analyzed public sentiment values from the internet in 2018 and 2019. Interestingly, the sentiment values in the previous two years did not demonstrate a downward trend in the second half of the year, indicating that the downward trend is probably not a result of common seasonal fluctuations (Figure 2a). It is worth noting that we observed three peaks of public sentiment values in week 1, week 7, and week 40, which were around the New Year, Spring Festival (ie, Chinese New Year), and the National Day, respectively, indicating the effects of holidays on public sentiment on the internet. The peak around the Spring Festival period observed in the previous two years did not occur in 2020. As the Chinese New Year was just after the COVID-19 outbreak, one might suspect that the negative effects of the pandemic were diluted by its holiday effects. To obtain a more accurate reading of the effect of the pandemic on public sentiment on the internet, it is necessary to exclude these holiday effects. Therefore, we estimated the holiday effects by using data of the previous two years. In particular, we computed the difference between sentiment values in the holiday week and those in the week before and after each holiday. Thus, the holiday effects were excluded from the sentiment values in 2020 for further analysis (Figure 2b).

According to the white paper published by the State Council Information Office of the People's Republic of China, titled “Fighting COVID-19 China in Action” [24], China divided its fight against the pandemic into five stages. The variations of sentiment values in 2020 were well aligned with these five stages of outbreak prevention. In stage 1 (weeks 0-3: *swift response to the public health emergency*), the sentiment values had been relatively stable given the pandemic had not yet triggered nationwide attention. In stage 2 (weeks 3-8: *initial progress in containing the virus*), due to public concern about the pandemic outbreak, the sentiment values rapidly declined and then rebounded quickly as the government brought the pandemic under control. In stage 3 (weeks 8-11: *newly confirmed domestic cases in mainland China decline to single digits*), sentiment values were restored to a stable level as the number of confirmed COVID-19 cases gradually decreased to single digits. In stage 4 (weeks 12-17: *Wuhan and Hubei—an initial victory in a critical battle*), sentiment values once again increased and even exceeded those in the same period in previous years. The sentiment values in stages 2-4 significantly correlated with the number of newly confirmed cases (Pearson correlation, *r*=–0.58, *P*=.02; see Figure 2b), indicating a strong correlation between public sentiment on social media and the severity of the pandemic.

However, in stage 5 (week 18 to present: *ongoing prevention and control*), there was another unexpected decline in the public sentiment values even though the pandemic was well under control at the time. Interestingly, public sentiment values in this stage were no longer correlated with the number of newly confirmed COVID-19 cases (*r*=0.12, *P*=.50).

### Relationship Between the Decline in Sentiment Values in Stages 2 and 5

**Figure 3:**
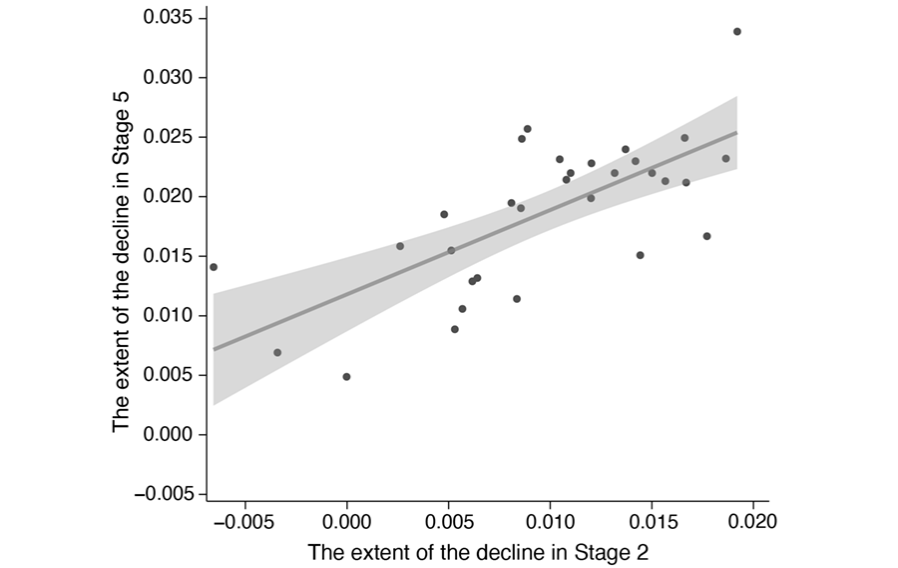
A correlation between the declines observed in social media public sentiment values in stage 2 with those in stage 5. Black dots represent the extent of the decline of public sentiment values in each province in mainland China. The solid line represents the fitted linear curve of the extent of the decline in two stages (*R^2^*=.50, *P*<.001, 95% CI 0.473-0.978).

In this study, we considered the underlying reasons for the decline in public sentiment on social media in stage 5. We hypothesized that the decline in sentiment values could be an after-effect of the decline in public sentiment in stage 2. Considering the different depths of the pandemic’s severity across China, the extent of the decline in public sentiment values similarly varied across different provinces and municipalities. We reasoned that if there was a correlation between the extent of the decline in public sentiment values in stages 2 and 5, noting that it is likely that the decline in stage 5 reflects a long-term consequence of the outbreak in stage 2. To test the hypothesis, we calculated the difference of sentiment values between week 3 and 5 as the extent of the decline in stage 2 and the difference of sentiment values between weeks 17 and 46 as the extent of the decline in stage 5. We observed a significant positive correlation between the extent of the decline in stage 2 and that in stage 5 (*r*=0.71, *P*<.001), which supports our hypothesis that the decline in public sentiment values in stage 5 reflects the long-term emotional consequences induced by the initial COVID-19 outbreak (Figure 3).

### Alternative Causes Leading to the Decline in Public Sentiment on Social Media in Stage 5

#### Concerns About Upgrading Risk Assessment Level

**Figure 4:**
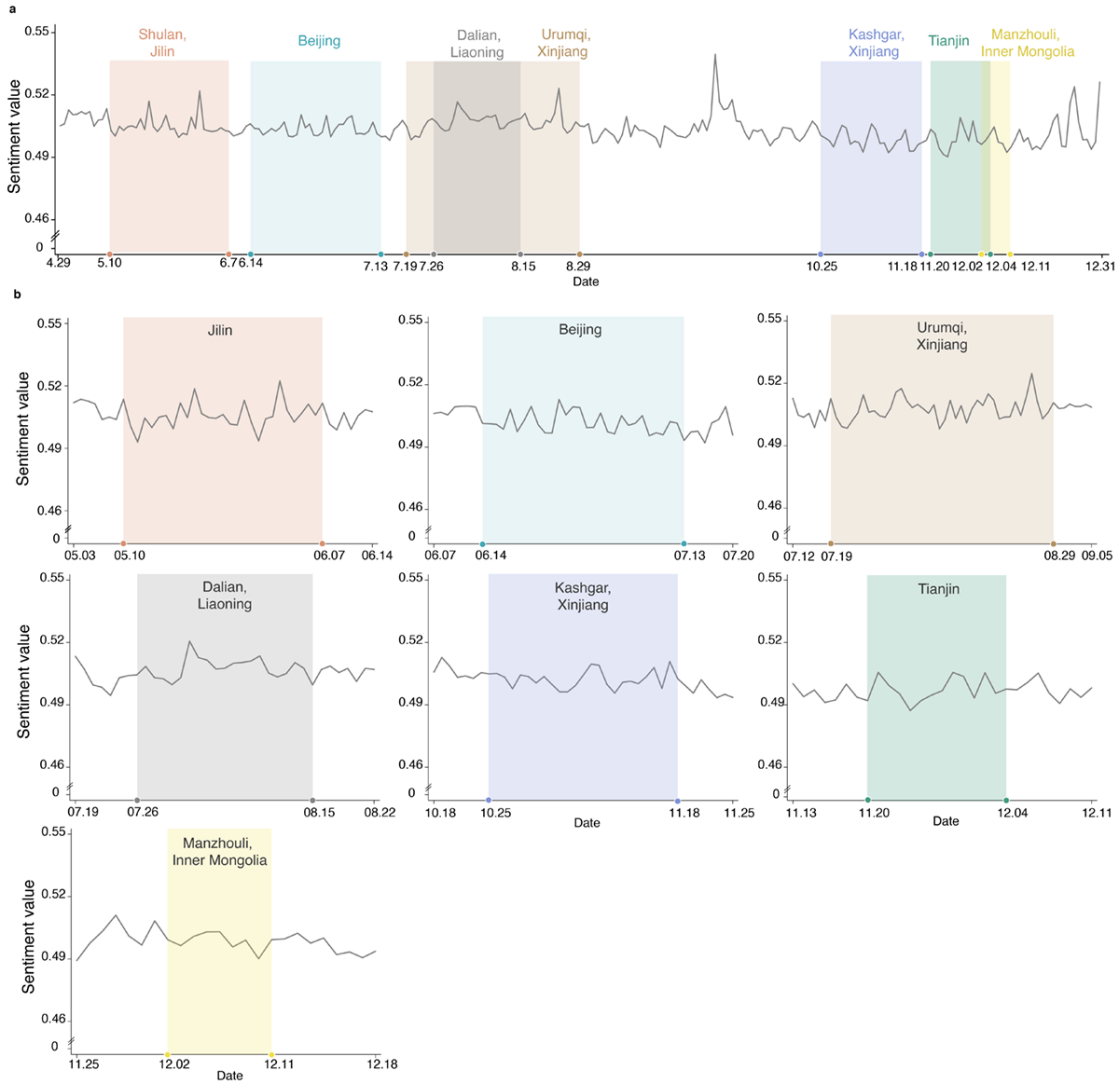
The effect of sporadic indigenous inflection cases in stage 5. (a) National sentiment values in stage 5. The time windows were labeled with underlay colors when the risk assessment level was upgraded to high risk in some areas. (b) Local sentiment values in the provinces when the local risk assessment levels were upgraded.

Sporadic indigenous COVID-19 cases remained in stage 5. In response to the emergence of these local cases, governments upgraded the risk assessment level from *low* to *high* in Jilin (weeks 19-23), Beijing (weeks 24-28), Xinjiang (weeks 29-35), Liaoning (weeks 30-33), Xinjiang (weeks 43-47), Tianjin (weeks 47-49), and Inner Mongolia (weeks 49-50). Upgrading the risk assessment level may have induced negative emotions in the general public. To assess whether that was indeed the case, we defined average public sentiment values 1 week before and after the high-risk period as the baseline. We defined the rate of change by the difference between the average public sentiment values in the high-risk period and the baseline, then divided by the baseline. The rate of change was close to 0 either at the nationwide scale (Jilin: –0.19%, Beijing: 0.18%, Xinjiang: 0.94%, Liaoning: 0.61%, Xinjiang: –0.18%, Tianjin: 0.28%, Inner Mongolia: –0.21%; see Figure 4a) or local scales (Jilin: –0.13%, Beijing: –0.44%, Xinjiang: 0.17%, Liaoning: 0.57%, Xinjiang: 0.20%, Tianjin: 0.21%, Inner Mongolia: 0.05%; see Figure 4b).

#### Concerns About the Economic Consequences of the COVID-19 Pandemic

**Figure 5:**
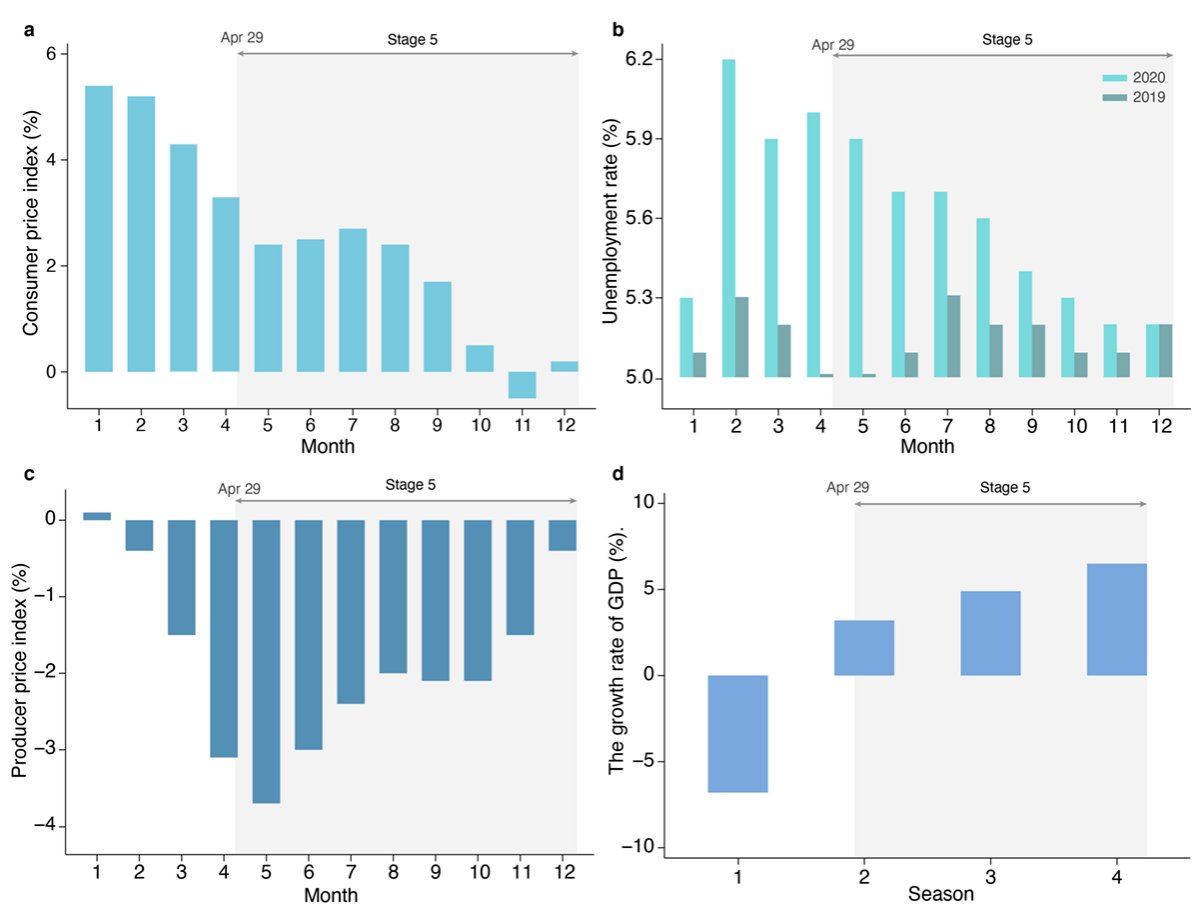
Major economic indicators in 2020 in mainland China, including (a) consumer price index, (b) unemployment rate, (c) producer price index, and (d) gross domestic product (GDP) growth rate.

In stage 5, although the number of newly confirmed COVID-19 cases was relatively lower than earlier in the year, it induced negative effects on economic activities. These negative socioeconomic impacts could lead to public pessimism about economic situations. To test the hypothesis, we computed several social and economic indicators, such as consumer price index, unemployment rate, producer price index, and growth rate of GDP. The consumer price index had fallen since March and was in line with the previous year’s level by the end of 2020 (Figure 5a). The unemployment rate also fell to 5.2 at the end of the year, which was lower than the high point of 6.2 at the beginning of the year (Figure 5b). Moreover, the unemployment rate in December was close to that in the previous years. Since February, the producer price index was lower than that in the corresponding period in the last year owing to the impact of the pandemic. However, the gap narrowed after May and reached about 0.4% in December of 2020 (Figure 5c). Meanwhile, the GDP increased, reaching a growth rate of 3.2%, 4.9%, and 6.5% in quarters 2, 3, and 4, respectively (Figure 5d). All major economic indicators showed largely positive economic trends in mainland China in stage 5, leading us to speculate that the decrease in social media public sentiment indices was probably not because of concerns about the impact of economic factors.

#### Concerns About the Severity of the Global Pandemic

**Figure 6:**
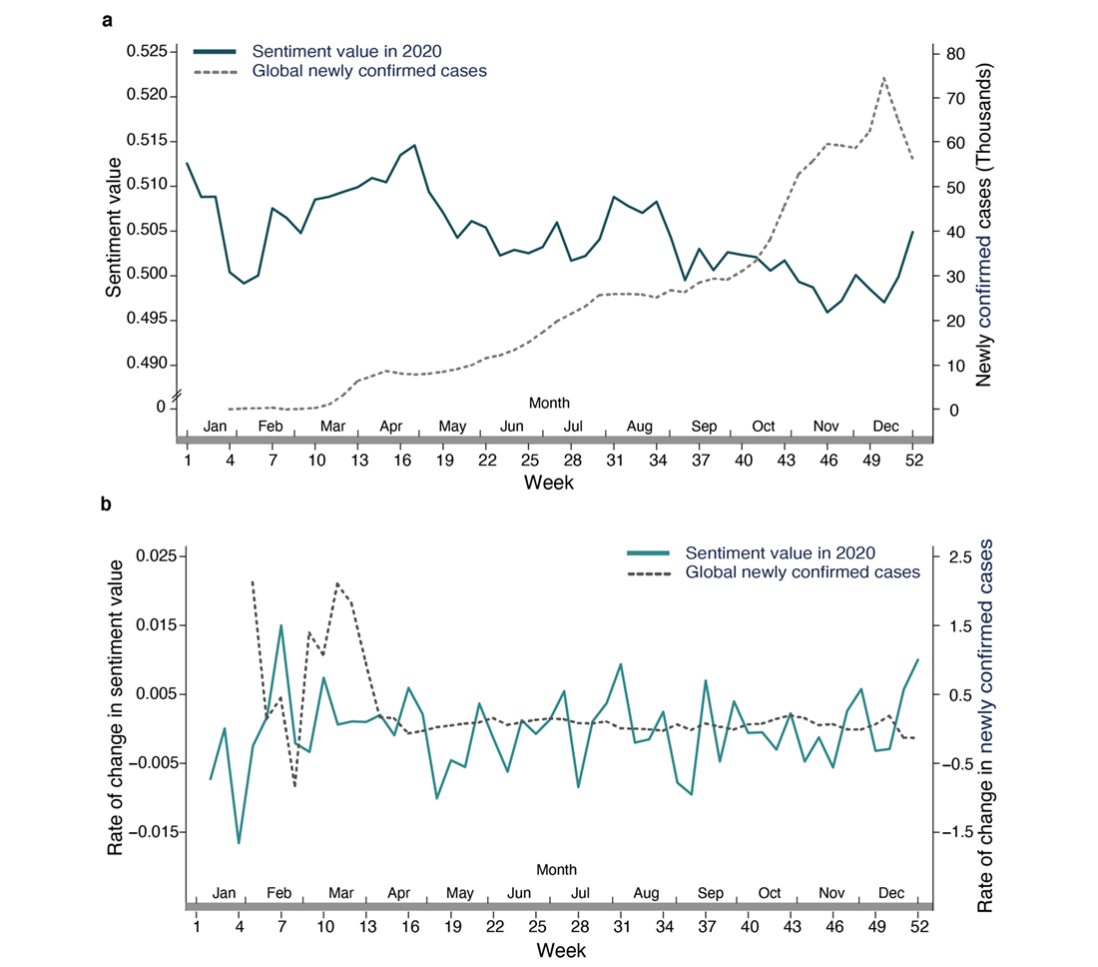
Comparisons of (a) global newly confirmed COVID-19 cases with social media public sentiment values and (b) the rates of change between newly confirmed cases globally and sentiment values.

In stage 5, although mainland China entered a recovery period, the global pandemic had been worsening. This led to considerations of whether the decline in public sentiment on social media during stage 5 was due to the increasing severity of the global pandemic. To answer this, we compared the sentiment values with the number of newly confirmed COVID-19 cases worldwide in stage 5. Although newly confirmed cases increased while sentiment values decreased in stage 5, timelines of global newly confirmed cases of COVID-19 and sentiment values were out of sync. We calculated the rate of change of newly confirmed cases globally and sentiment values by taking weekly data points. We first subtracted the data point 1 week earlier and then divided the value by the data point 1 week earlier. We observed the rate of change of newly confirmed cases globally and sentiment values did not match (*r*=0.03, *P*=.85; see Figure 6b). This analysis indicates that the severity of the global pandemic may not be the reason for the decline in social media public sentiment values in China during stage 5.

## Discussion

### Principal Findings

This study tracks public sentiment on social media in mainland China throughout the year of 2020 across the five official stages of the COVID-19 pandemic—from *city lockdown* to *the new normal*. Such uninterrupted long-term tracking allows us to obtain a comprehensive picture of the pandemic's impact on public sentiment on social media. Moreover, we analyzed the data from the corresponding period in the previous two years, while excluding the influence of other possible confounding factors such as holidays. Our analyses identified an interesting new phenomenon—a double descent of public sentiment on social media during the pandemic. The first descent verifies a strong negative effect of public sentiment at the outbreak of COVID-19. More importantly, the second descent illustrates the impact of the pandemic on public sentiment on social media during the recovery stage, which has not been previously reported in the literature.

In terms of the relationship between public sentiment on social media and the severity of the pandemic, there are two separate phases with the boundary at week 17. The first phase, week 1 to week 17, covers stages 1 to 4, defined by the white paper published by the Chinese government. Our study corroborates previous studies, validating that public sentiment was strongly affected by the outbreak of COVID-19 and it was correlated with the severity of the pandemic [33-35]. Moreover, we observed that the public sentiment values increased as China brought the pandemic under control. Especially when the city of Wuhan was reopening, the public sentiment on social media index was even higher than that in the same period in the previous two years, showing a highly positive emotion among the general public after the recovery from the pandemic [16]. These consistencies support that the sentiment analysis of Weibo posts accurately reflects public sentiment during the pandemic.

Public sentiment after week 17, which was defined as stage 5 in the Chinese government's white paper on epidemic control, has not been investigated in previous studies. In this stage, the government had brought the spread of the pandemic under control and socioeconomic life had recovered substantially. We observed a surprisingly sustained decline in social media public sentiment values in stage 5, which differs from the decline in stage 2 in several aspects. First, the public sentiment values in stage 5 were decoupled from the severity of the pandemic. Second, the decline observed in stage 2 lasted only 3 weeks, whereas the decline in stage 5 lasted for at least 34 weeks, with a much slower reduction rate. Third, the social media public sentiment values in stage 5 decreased as a descending spiral rather than a straight line. These characteristics indicate that the decline in stage 5 is unlikely to be a real-time reaction induced by a single event, instead of reflecting a long-term emotional trend in the general public.

### Cause Analysis

The underlying causes of such a prolonged decline in public sentiment in stage 5 need to be evaluated. After excluding possible economic reasons and comparing the extent of reduction between public sentiment values in stage 2 and stage 5, we can speculate that the decline could be a long-term effect of the emotional shock of the COVID-19 outbreak. Psychological studies have shown that people exposed to a traumatic event, such as an earthquake, tsunami, or terrorist attack, often struggle with symptoms of posttraumatic stress disorder (PTSD) [36,37]. It is expected that a severe pandemic such as COVID-19 would induce PTSD among many groups, including infected patients [38,39], medical workers [40,41], people related to them, and those who are forced into isolation [42]. However, it is somewhat surprising that there is such a strong and long-last negative effect of the sentiment among the general population. This finding reflects the far-reaching public psychological impact of this unprecedented pandemic.

### Limitations and Future Research

Despite the efficiency of using social media for analyzing public sentiment on social media, there remains a gap between the sentiment obtained from texts on the web and real emotion in the general population. Such a gap derives from the sampling bias between the users of the microblogging network and the population in the social group. Although internet access has become widespread in China and Weibo is the largest social media platform there, Weibo users do not represent an unbiased sample of the overall Chinese population because the population of internet users is relatively young and concentrated in metropolitan centers [43]. Moreover, there may also be a difference between the emotions people write in public media and their internal emotions. At the same time, our findings are based on Chinese social media data. At the current stage, mainland China is the only major world economy that has experienced a relatively complete cycle from early outbreaks to the recovery of socioeconomic activities. It is unclear how public sentiment changes in other regions when they enter the recovery phase of the pandemic.

Weibo’s data reflect people's emotions in public scenarios. People may not express their emotions freely to the public due to various concerns. In contrast, the private messages among friends could reflect their internal emotions. In China, there is a popular application named WeChat, which provides text messaging and broadcast messaging among friends. Analysis of WeChat messages may provide public emotions from the other perspective. Moreover, comparing the data of Weibo and WeChat would also show the difference in people's emotional expressions between public and private scenarios. However, WeChat messages are private data and not publicly available, and it would not meet the ethical requirements of using data without the permission of account owners. It would be very valuable to analyze WeChat data if there is a solution to use the data without ethical violations and invasion of personal privacy.

The long-term psychological effects of the COVID-19 pandemic are going to be quite complex and far reaching. Further research will track the effects of the epidemic in the scale of years. Meanwhile, with increasing immunization rates, the epidemic has been sufficiently under control in many other countries. It would be interesting to compare the public emotions across countries with the different cultures after the pandemic. Moreover, given the fact that different countries implement different policies to prevent the spread of the pandemic, it is important to analyze the effects of social policies on public sentiments. These studies would provide very useful information for psychological interventions and social warnings in the post-epidemic world.

### Conclusions

In summary, by tracking public sentiment on social media for the whole year of 2020, we were able to evaluate the long-term negative impact of COVID-19 on public sentiment, which shows the complexity and far-reaching impact of the pandemic on human emotions. This study’s results suggest that, from a public policy perspective, even when the pandemic has been controlled and socioeconomic activation is restored, decision-makers must still pay attention to public sentiment and take necessary action to alleviate the negative emotions induced by the pandemic.

## Abbreviations

GDP: gross domestic product
SARS: severe acute respiratory syndrome
